# The Work Done by the Human Heart

**Published:** 1870-04

**Authors:** 


### The Work Done by the Human Heart.

From a long article (by Professor Haughton, of Dublin,) in a recent number of *Nature,* we condense the following interesting facts:—

The total daily work of the human heart is equivalent to 124,208 tons (of 2,240 pounds each) lifted one foot.

The daily labor of a workingman, deduced from long continued observation of various kinds of labor, is found to be equal to 354 tons lifted through one foot, during the ten hours. This is less than three times the work done by a single heart, beating day and night for twenty-four hours; so that three old women sitting beside the fire, alternately spinning and sleeping, do more work by the constant beating of their hearts than can be done in a day by the sturdiest farm laborer,

In a boat race, it is calculated that fifteen foot-pounds of work are performed by each ounce of muscle during each minute of the rowing. No muscular labor that man can undertake is more severe than this; and yet this labor is only three-fourths of that which is exerted day and night during life by each of our hearts.

If the heart should expend its entire force in lifting its own weight vertically, it could raise that weight 19,754 feet in an hour. An active pedestrian can climb from Zermatt to the top of Monte Rosa, 9,000 feet, in nine hours; or can lift his own body at the rate of 1,000 feet an hour, which is only one-twentieth part of the energy of the heart.

When the railway was built from Trieste to Vienna, a prize was offered for a locomotive engine that could lift its own weight through the greatest height in one hour. The "Bavaria," which won the prize, lifted itself through 2,700 feet in an hour. This is the greatest feat yet accomplished on steep grades, and is considered very remarkable; but it is only one-eighth part of the mechanical force of the heart.

The heart, then, is the most wonderful of machines. Its energy equals one-third of the total daily force of all the muscles of a strong man; it exceeds by one-third the labor of the muscles in a boat-race, estimated by equal weight of muscle; and it is twenty times the force of all the muscles used in climbing, and eight times the force of the most powerful engine which the art of man has yet invented.—*Journal of Chemistry*.

